# Impact of oleic acid as co-substrate of glucose on “short” and “long-term” Crabtree effect in *Saccharomyces cerevisiae*

**DOI:** 10.1186/1475-2859-12-83

**Published:** 2013-09-23

**Authors:** Jillian Marc, David Feria-Gervasio, Jean-Roch Mouret, Stéphane E Guillouet

**Affiliations:** 1 Laboratoire d'Ingénierie des Systèmes Biologiques et des Procédés (LISBP), Institut National des Sciences Appliquées (INSA), UMR INSA/CNRS 5504, UMR INSA/INRA 792, 135 Avenue de Rangueil, 31077 Toulouse Cedex 4, France

**Keywords:** *Saccharomyces cerevisiae*, Ethanolic fermentation, Oleic acid, Crabtree effect, Accelerostat, Batch, Fed-batch

## Abstract

**Background:**

Optimization of industrial biomass directed processes requires the highest biomass yield as possible. Yet, some useful yeasts like *Saccharomyces cerevisiae* are subject to the Crabtree effect under glucose excess. This phenomenon can occur in large scale tank where heterogeneities in glucose concentrations exist. Therefore yeasts encounter local environments with glucose excess leading to ethanol production to the detriment of biomass formation. We previously demonstrated that oleic acid as a co-substrate in glucose-limited chemostat allowed to delay and modulate the “short-term” Crabtree effect in *Saccharomyces cerevisiae*. Here we further investigated the effect of oleic acid as a modulator of the Crabtree effect.

**Results:**

The impact of oleic acid as co-substrate on the Crabtree effect was investigated in terms of i) strain specificity, ii) reversibility of the potential effect with aerobic glucose-excess batches and iii) durability and maximal capacities under high ethanol stress with glucose-excess fed-batches. First, the addition of oleic acid resulted in an increase of the critical dilution rate by 8% and the specific carbon uptake rate by 18%. Furthermore, a delay was observed for the onset of ethanol production when a batch was inoculated with cells previously grown in glucose-oleate chemostat. Finally, the culture of adapted cells in a glucose-oleate fed-batch led to a redirection of the carbon flux toward biomass production, with a 73% increase in the biomass yield.

**Conclusions:**

This work demonstrated clearly that the perturbation by oleic acid as co-substrate resulted in a decrease in the “short-term” and “long-term” Crabtree effects. This impact was not strain dependent and reversible. Thus, industrial applications of this biochemical strategy could be envisaged to tackle heterogeneities issues in large scale tanks or to prepare starter yeasts for various applications.

## Background

The repression of oxidative pathways by glycolytic activity resulting in simultaneous fermentation and respiration was named “Crabtree effect” [1-3]. The resulting oxido-reductive metabolism is characterized by a byproduct formation (ethanol, glycerol and weak acids), a loss in biomass yield and a limited oxygen specific uptake rate. It is named “long-term” effect when it appears in glucose excess batch and fed-batch or in glucose-limited chemostat at dilution rates over the critical dilution rate (D_c_), involving adaptation of cell metabolism, while it is named “short-term” effect after a glucose pulse in glucose-limited chemostat at dilution rates under D_c_[4].

The mostly global accepted theory to explain this mechanism is a limited respiratory capacity in Crabtree-positive cells. This respiratory capacity includes the whole cytosolic and mitochondrial pathways responsible for the respiratory catabolism of pyruvate, including the respiratory chain. It is saturated above a critical threshold of glucose uptake rate. Then, the cell directs the excess of glycolytic flux toward fermentative metabolism with apparition of ethanol production [5,6]. This theory was supported by studies using genetic engineering approach. However, isolated deletion or over-expression of various genes coding for enzymes within the respiratory capacity did not success to bring out any single limiting reaction [7-15].

Modulation of the metabolic transition was successfully reported after engineering global regulation functions. The deletion of *MIG1* and *MIG2*, coding for a positive regulator in glucose repression led to a 5% higher D_c_, at 0.274 h^-1^[16]. The over-expression of *HAP4*, coding for a positive regulator of genes involved in respiratory metabolism, led to a 10% higher D_c_, at 0.33 h^-1^[17]. Decreasing intracellular NADH/NAD^+^ ratio by expressing a heterologous gene coding for a mitochondrial oxidase (*AOX1*) led also to a 10% higher D_c_, at 0.32 h^-1^[18]. Furthermore, modulation of “short-term” Crabtree effect was also reported through co-substrate feeding. Feeding oleic acid as a co-substrate of glucose enabled to delay and modulate the “short-term” metabolic transition apparition in cells [19].

The latter approach was carried out by feeding an aerobic glucose-limited chemostat at D = 0.16 h^-1^ with oleic acid. After a glucose pulse of 10 g L^-1^, a delay up to 15 min in the onset of the metabolic shift, a 33% decrease in the ethanol production, and a redirection of the carbon flux toward biomass production were observed. Moreover, specific activities of carnitine-acetyl transferase, isopropylmalate synthase and citrate synthase were increased. These ones are enzymatic systems involved in the transport of acetyl-coA through the mitochondria membrane [20-24]. Nevertheless, explanation of oleate effect on the metabolism remained unclear. First, the presence of this acid as co-substrate of glucose may have induced a “substrate effect” (degradation of the oleic acid through the β-oxidation pathway introducing a supplemental carbon flux into the central metabolism with potentially allosteric modifications). Feria-Gervasio et al. [19] conducted a glucose pulse experiment during a glucose-succinate chemostat in order to mimic the substrate effect of the oleic acid, but data obtained suggested that the “substrate effect” was not the major effect responsible for the decrease in the transition from respiratory to fermentative metabolism. Secondly, oleic acid may have induced a “genetic effect”, acting at the transcriptional and / or translational level *via* regulatory complex systems. Indeed, this acid is known to induce the transcription of various genes possessing the ORE (Oleate Response Element) sequence on their promoter [23]. This last hypothesis was strengthened by the observation of increased specific activities of enzymes coded by such genes in presence of oleic acid [19].

These previous efforts led to an overview of the impact of oleic acid on the “short-term” Crabtree effect. Nonetheless, the processes used for industrial applications for which Crabtree effect is unwanted, like starter yeasts or heterologous protein production, could led to “long-term” as well as “short-term” Crabtree effect. Moreover, the range of *S*. *cerevisiae* strains used in industry is wide and their behavior could largely differ from CEN.PK 113-7D. Based on these observations, previous work of Feria-Gervasio *et al*. [19] needed to be completed. Thus in the present work, we further investigated the impact of oleic acid including a determination of its specificity using an industrial strain (*S*. *cerevisiae* CA10/pCD63), the determination of the critical dilution rate for purely oxidative metabolism (D_c_), an investigation of the reversibility of the effect and finally a characterization of the durability of the effect under conditions resulting in “long-term” Crabtree effect onset.

## Results & discussion

We previously demonstrated that oleic acid as a co-substrate in glucose-limited chemostat (D=0.16 h^-1^) allowed to delay and modulate the transition from respiratory to oxido-reductive metabolism, so called “short-term” Crabtree effect, in *Saccharomyces cerevisiae* CEN.PK 113-7D. As a consequence of the modulation of the transition, ethanol production was decreased and the carbon flow was redirected toward the biomass production [19]. In order to further characterize this effect, the impact of oleic acid was investigated in terms of strain specificity, durability and reversibility of the effect.

### The oleic acid impact was not specific to the CEN.PK 113-7D strain

**Figure 1:**
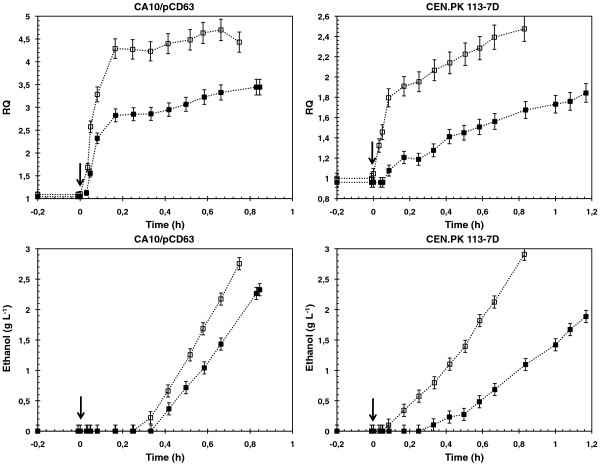
**Changes in the ethanol concentration and RQ upon a glucose pulse.** Pulse of 10 g L^-1^ of glucose (indicated by the *arrow*) was done at the steady state of glucose limited chemostat of strain CA10/pCD63 and CEN.PK 113-7D in absence (*open symbols*) or in presence (*full symbols*) of an oleic acid feeding at D_ole_ = 0.0041 h^-1^ (CA10/pCD63) and D_ole_ = 0.0073 h^-1^ (CEN.PK 113-7D). Carbon balances were evaluated on the totality of each glucose pulse experiment within the range 100 ± 2%.

To evaluate the strain specificity of the impact of oleic acid on the transition from oxidative to oxido-reductive metabolism, glucose pulse experiment was carried out at the steady state of a glucose-limited chemostat with and without oleic acid with the strain CA10/pCD63. Results were compared with those previously obtained with *S*. *cerevisiae* CEN.PK 113-7D. Figure 1 showed the evolutions of the ethanol concentration and the Respiratory Quotient (indicative of the ratio between the utilization of fermentative and oxidative pathways) after a glucose pulse at 10 g L^-1^ for the two strains. The presence of oleic acid in the chemostat of *S*. *cerevisiae* CA10/pCD63 led to a decrease in ethanol (16%) and acetate (results not shown) production after the glucose pulse. The RQ profile was also lower in presence of oleic acid and confirmed the lower utilization of fermentative pathway in this condition. Its maximal value was decreased by 27% and 25% respectively for CA10/pCD63 and CEN.PK 113-7D. Finally, the strain CA10/pCD63 showed a delay of 6 minutes in the onset of the metabolic shift upon the glucose pulse (calculated as the difference between the first data points where ethanol was detected in the cultures with and without oleic acid). The delay was found equal to 15 min for the strain CEN.PK 113-7D under the same conditions.

Thus, the impact of oleic acid on the dynamic profiles of ethanol production and RQ were similar for both strains, however less slightly pronounced for *S*. *cerevisiae* CA10/pCD63.

### Oleic acid impacted also the critical dilution rate (D_c_)

In order to determine the critical dilution rate for S. *cerevisiae* CEN.PK 113-7D with and without oleic acid as co-substrate (D=0.0073 h^-1^), accelerostat cultures were carried out by increasing the glucose feeding as a function of time. The dilution rate was increased from an initial value of 0.16 h^-1^ to a final value of 0.30 h^-1^ with a constant acceleration of 0.005 h^-2^.

**Figure 2:**
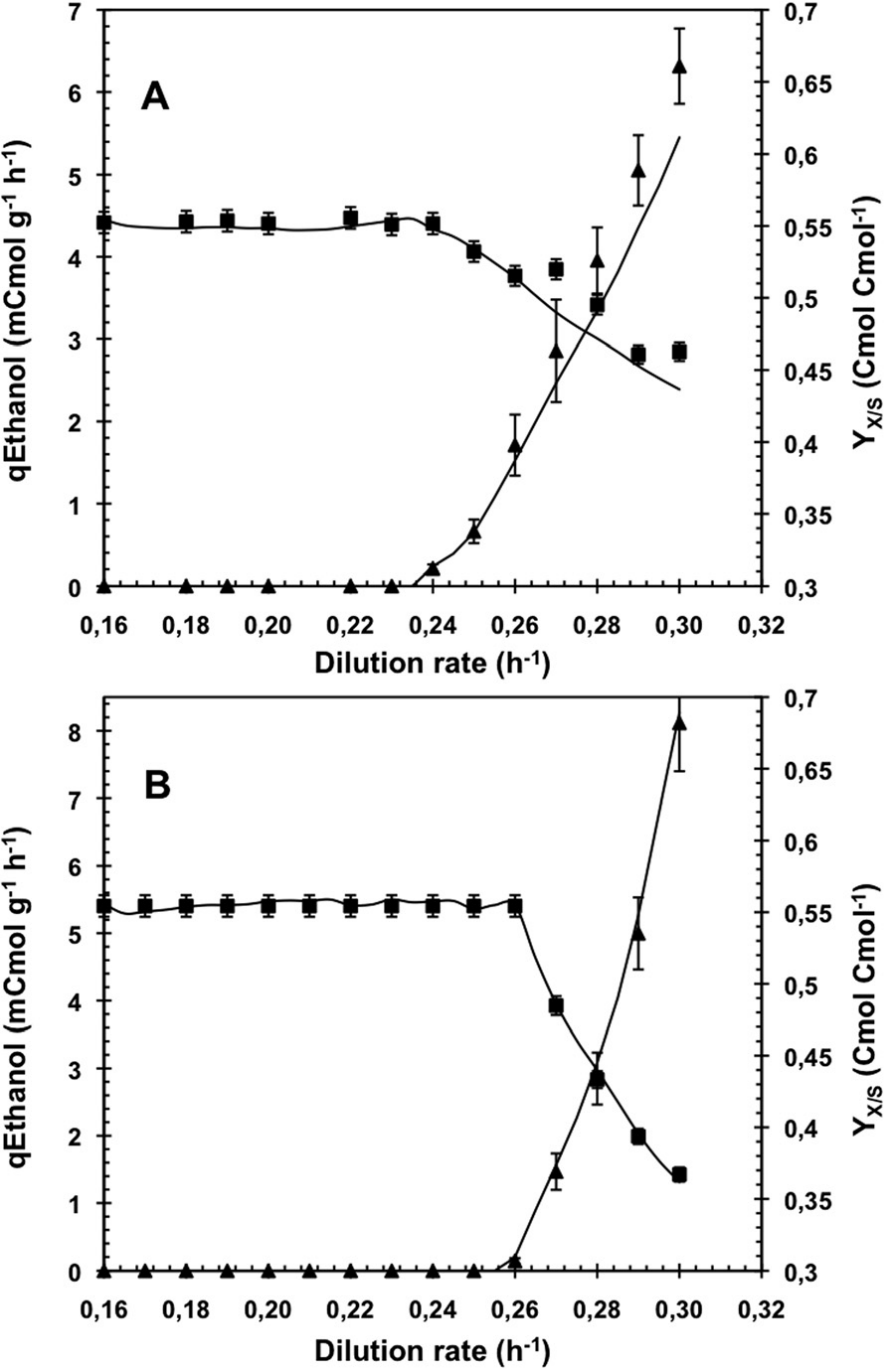
**Specific ethanol production rate and biomass yield during A**-**stat cultivations.** Specific ethanol production rate (▲) and biomass yield (■) during A-stat cultivations on glucose in absence **(**AS_G_C_G_; **A)** or presence **(**AS_GO_C_GO_; **B)** of oleic acid feeding at D_ole_ = 0.0073 h^-1^. *Lines* represented the evolution profile calculated by carbon and degree of reduction balances.

The onset of the transition from respiratory to oxido-reductive metabolism, corresponding to the D_c_, was characterized by the increase in the ethanol production and the decrease in the biomass yield (Y_x/s_). This happened at 0.24 h^-1^ on sole glucose (Figure 2A) but the presence of oleic acid in the fermentation broth led to an 8% increase at 0.26 h^-1^ (Figure 2B). The difference in D_c_ was in the same order of magnitude as those found in the literature with mutant strains. A first study deleting *MIG1* and *MIG2*, coding for a regulator involved in the glucose repression, led to a 5% increase in the D_c_ determined with the same technique [16]. Over-expressing the transcription factor Hap4p led to a 10% increase in D_c_ estimated in chemostat cultures [17]. Finally, Vemuri *et al*. [18] showed a 10% increased in D_c_ by expressing a heterologuous oxidase during productostat cultures [25].

**Figure 3:**
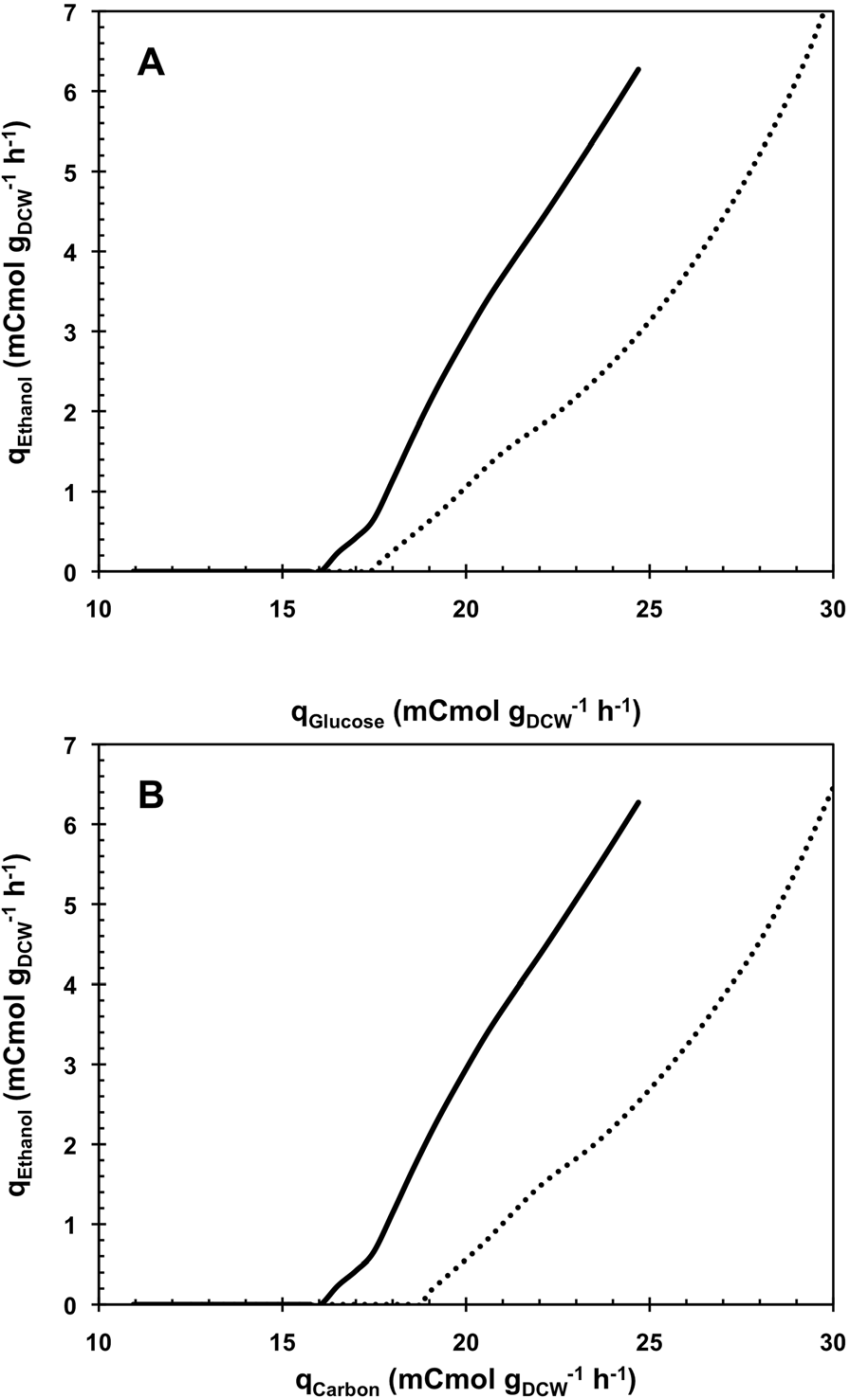
**Specific ethanol production rate as a function of the specific consumption rates of glucose and carbon during A**-**stat cultivations.** Specific ethanol production rate is given as a function of the specific consumption rates of glucose **(A)** and the specific consumption rate of carbon **(B)** during A-stat cultivations on glucose in absence (AS_G_C_G_; *solid lines*) or presence (AS_GO_C_GO_; *dotted lines*) of oleic acid feeding at D_ole_ = 0.0073 h^-1^. The A-stat cultivations of *Saccharomyces cerevisiae* CEN.PK 113-7D were carried out between D = 0.16 h^-1^ and D = 0.3 h^-1^.

Under cellular metabolism considerations, this 8% increase in D_c_ corresponded to an 8% higher specific consumption rate of glucose for the onset of the metabolic transition, from 16.5 to 17.8 mCmol gDCW^-1^ h^-1^ (Figure 3). Previous study of Feria-Gervasio *et al*. [19] demonstrated that modulation of this transition was not correlated to the electron transport chain capacity. Thereby, both observations suggested that addition of oleic acid resulted in an enhancement of the so-called “respiratory capacity” (black box model including the metabolic cytosolic and mitochondrial pathways responsible for the respiratory catabolism of pyruvate), permitting cells to metabolize glucose at higher rates without employing the fermentative way.

In regard to the total carbon (glucose and oleic acid) uptake rate, the D_c_ corresponds to a 15% higher carbon uptake rate in glucose-oleate A-stat, from 16.5 to 19.0 mCmol gDCW^-1^ h^-1^ (Figure 3), oleic acid carbon accounting for 7%.

### Oleic acid effect was at least partially reversible

Batch cultures were performed in order to determine the reversibility of the oleate impact. Pre-grown cells on glucose-oleate carbon source were transferred in glucose batch cultures with or without oleic acid as co-substrate (B_G_C_GO_ and B_GO_C_GO_). Their ethanolic performances were compared to a control glucose batch with cells precultured on glucose only (B_G_C_G_).

**Figure 4:**
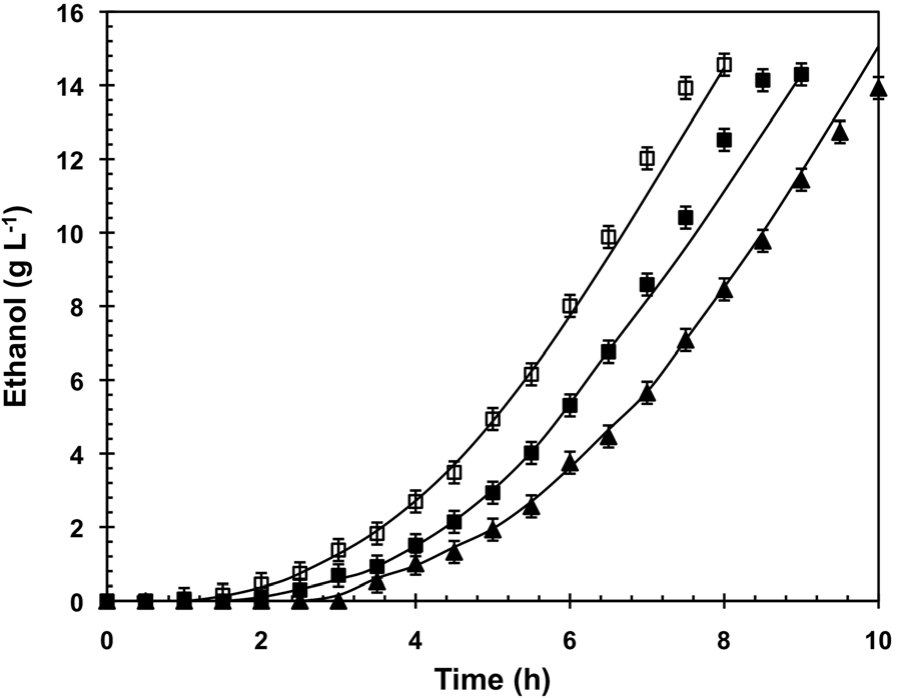
**Concentration of ethanol during aerobic batch cultivations.** The batch cultivations of *Saccharomyces cerevisiae* CEN.PK 113-7D were inoculated with cells gathered at the steady state of glucose limited chemostat at D=0.16 h^-1^ (Batch glucose (B_G_C_G_); ☐) or with cells gathered at the steady state of glucose limited chemostats at D=0.16 h^-1^ in presence of oleate at Dole=0.0073 h^-1^ (Batch glucose (B_G_ C_GO_); ■ and Batch glucose with oleic acid (B_GO_ C_GO_); ▲). *Lines* represented the evolution profile calculated by carbon and degree of reduction balances.

Batch cultivations on glucose only showed that cells precultured on glucose + oleic acid as the carbon sources (B_G_C_GO_) presented a 1 hour greater delay in the onset of ethanol production than the cells pregrown on glucose only (B_G_C_G_) (Figure 4). Biomass-oriented rearrangement of the metabolism was thus conserved even when oleate was removed from the culture broth. However, cells precultured on glucose + oleic acid as the carbon sources and transferred in a batch containing glucose and oleic acid (B_GO_C_GO_) presented a 1 hour even greater delay than when growing on glucose alone (B_G_C_GO_). As a consequence, a constant oleic acid pressure seemed necessary to conserve for a long period the whole impact on the cell metabolism. Therefore, these observations indicated a partial reversibility of the oleic acid effect in cells once oleic acid was removed.

### Oleic acid impact was effective for hours during VHEP fermentation under oleate pressure

The impact of oleic acid on the oxido-reductive metabolism and its durability were further studied during fed-batch conditions. A first glucose-excess aerobic fed-batch cultivation was carried out with glucose as sole carbon source inoculated with cells precultured on glucose (FB_G_C_G_). A second fed-batch was carried out with cells pregrown on glucose and oleic acid as co-substrate (FB_GO_C_GO_) for comparison.

**Figure 5:**
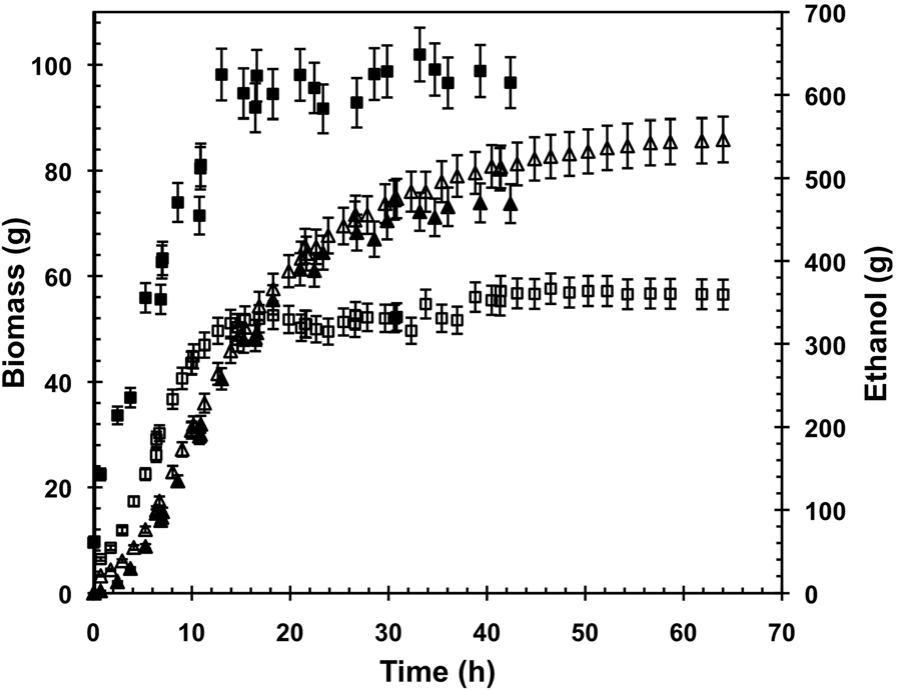
**Evolution of biomass and ethanol cumulated masses during aerobic fed**-**batch cultivations.** (☐) biomass and (△) ethanol produced by *Saccharomyces cerevisiae* CEN.PK 113-7D. *Open symbols* represent the glucose-excess fed-batch with non-adapted cells (FB_G_C_G_), *full symbols* represent the glucose-oleate-excess fed-batch with adapted cells (FB_GO_C_GO_).

Kinetics of biomass and ethanol produced suggested that the presence of oleic acid in the medium promoted the growth of yeasts to the detriment of ethanol production and led to a shorter fermentation time (Figure 5). This was confirmed in view of the global yields on glucose consumed (Table 1). Biomass yield was increased by 35% as well as its final concentration by 73%. Ethanol yield was meanwhile decreased by 8% and the final titer of ethanol by 7% (116.2 vs 126.8 g L^-1^), although its yield was not statistically different. The maximal production rates of ethanol and glycerol were decreased by 67% and 39%, respectively (Table 1). This clearly confirmed that oleic acid negatively impacted the fermentative capacities of the cells during aerobic fed-batch cultures. Carbon metabolism was thus preferentially directed toward biomass production although no variation was observed for the maximal specific growth rate.

**Table 1:** **Final concentrations**, **global yields and maximal specific rates obtained during aerobic fed**-**batch cultivations**

			**Biomass**	**Ethanol**	**Glycerol**	**Acetate**	**Succinate**	**Pyruvate**
**Final concentration**	**FB**_**G**_**C**_**G**_	**g L**^-**1**^	13.2 ± 0.3	126.8 ± 0.6	4.96 ± 0.02	5.28 ± 0.04	0.53 ± 0.01	0.36 ± 0.01
	**FB**_**GO**_**C**_**GO**_	**g L**^-**1**^	22.9 ± 0.5	116.2 ± 1.1	4.21 ± 0.05	5.1 ± 0.1	0.71 ± 0.03	0.37 ± 0.06
	**Difference**	%	**73**	-**8**	-**15**	3	**34**	3
**Yield**	**FB**_**G**_**C**_**G**_	**g g**^-**1**^	0.174 ± 0.005	0.432 ± 0.008	0.0142 ± 0.0012	0.0158 ± 0.0006	0.0027 ± 0.0006	0.0013 ± 0.0003
	**FB**_**GO**_**C**_**GO**_	**g g**^-**1**^	0.234 ± 0.012	0.40 ± 0.01	0.015 ± 0.002	0.0171 ± 0.0004	0.0045 ± 0.0002	0.0013 ± 0.0002
	**Difference**	%	**35**	-**7**	6	**8**	**67**	0
**Maximal specific rate**	**FB**_**G**_**C**_**G**_	**g g**^-**1**^**L**^-**1**^	0.35 ± 0.01	1.31 ± 0.05	0.074 ± 0.008	0.034 ± 0.004	0.007 ± 0.002	0.013 ± 0.004
	**FB**_**GO**_**C**_**GO**_	**g g**^-**1**^**L**^-**1**^	0.38 ± 0.03	0.43 ± 0.03	0.045 ± 0.008	0.047 ± 0.002	0.015 ± 0.003	0.017 ± 0.004
	**Difference**	%	8	-**67**	-**39**	**38**	**114**	30

*Saccharomyces cerevisiae* CEN.PK 113-7D was cultivated under glucose-excess (FB_G_C_G_) or glucose-oleate-excess (FB_G0_C_G0_) conditions. Representative differences are in bold.

Table 1 showed a greater production of succinate in the cells precultured on glucose + oleic acid (FB_GO_C_GO_). Final concentration, global yield and maximal specific rate of production of this organic acid were increased respectively by 34%, 67% and 114% when oleate was added in the fermentation broth. Measurements did not reveal any oleate consumption during the fed-batch FB_GO_C_GO_ experiment. Moreover carbon mass balance was found close to 100% taking into account only glucose as substrate, confirming that no oleic acid was consumed. Therefore, a succinate synthesis from the catabolism of oleic acid through the β-oxidation and glyoxylate pathways cannot be taken into consideration. The cycle of the tricarboxylic acids was then supposed to be the source of succinate excretion under fermentative metabolism with glucose as sole carbon source, as previously mentioned by Camarasa *et al*. [26]. Moreover, HCO_3_^-^ was known to inhibit numerous enzymes such as TCA cycle succinate dehydrogenase [27]. During our experiments, succinate was produced in the first phase of the fed-batches, corresponding to the slightly extended growth phase, corresponding when the highest rates of CO_2_ production occurred (results not shown). Assuming that succinic acid derived from the tricarboxylic acids cycle (TCA), the increased excretion of succinate could be interpreted as a higher activity of the TCA cycle. These observations were strengthened by the increased oxygen consumption observed in presence of oleic acid (data not shown) and previously described by Feria-Gervasio *et al*. [19] in aerobic chemostat.

Glycerol production was also decreased in presence of oleic acid. Final concentration and maximal specific rate of production of glycerol were decreased respectively by 15% and 39%. In addition to its role in the stress response, glycerol formation is known to ensure the cytosolic NADH reoxidation when oxido-reductive metabolism occurs for anabolic purpose [28]. The decrease in glycerol production was likely the result of the increase in the oxygen consumption rate observed in the presence of oleic acid. Similar correlation between glycerol formation and oxygen consumption rate were reported in the literature either by modulating the aeration regime [29], by reducing the glycerol production through the control of the RQ [30] or by metabolic engineering approaches [18,31].

Considering the differences on succinate, pyruvate, glycerol productions and on oxygen consumption, we can reasonably make the statement that the presence of oleic acid enhanced the global respiratory capacity in *S*. *cerevisiae*.

**Figure 6:**
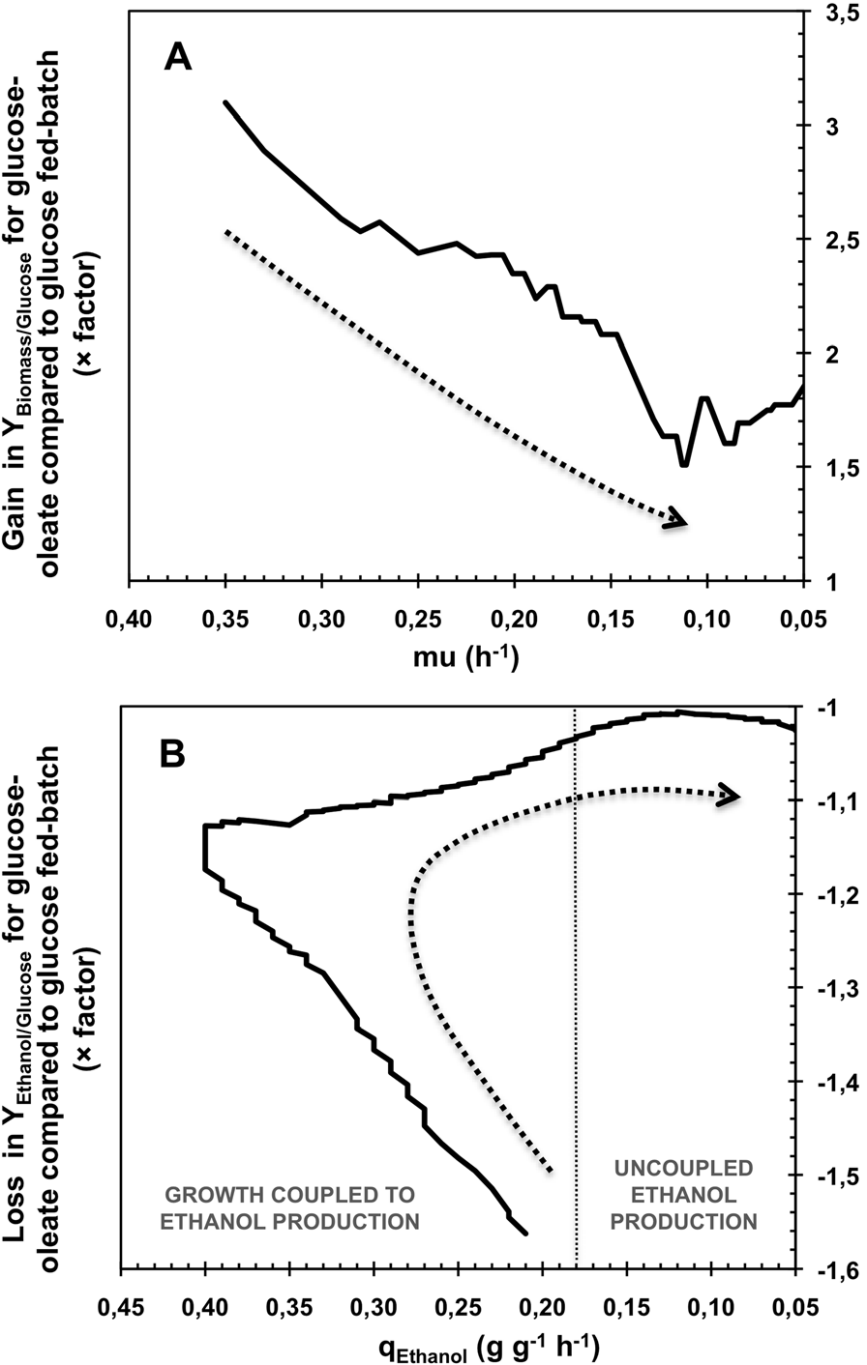
**Evolutions of the gain in biomass yield and the loss in ethanol yield during aerobic fed**-**batch cultivations.** Evolutions of the gain in biomass yield as a function of the growth rate **(A)** and the loss in ethanol yield as a function of the specific rate of ethanol production **(B)** during aerobic fed-batch cultivations of *Saccharomyces cerevisiae* CEN.PK 113-7D. The gain represents difference on yields obtained with glucose-oleate cultivation against glucose cultivation. Dotted arrows represent the fermentation progress.

Figure 6 presented the gain on biomass yield and the loss on ethanol yield in glucose-oleate fed-batch compared to glucose fed-batch. In these graphics, the diminution of μ and q_Ethanol_ can be linked to the experiment progress and permitted an overview on the evolution of the oleate impact strength on the cell metabolism. Gain on biomass yield decreased during the major part of growth phase, from an initial factor of 3.2 to a lower of 1.5. Moreover, loss on ethanol yield decreased from a factor of 1.57 at the beginning of the culture to 1.04 at the end of the growth phase, i.e. for q_Ethanol_ below 0.18 g g^-1^ h^-1^. When uncoupled ethanol production occurred, i.e. for specific rate of ethanol production below 0.18 g g^-1^ h^-1^, this factor was between 1 and only 1.04. This clearly revealed a continuous loss of the strength of the oleic acid impact on the cell metabolism with the culture progress. When the growth phase ended, this impact even almost disappeared. Thus, oleic acid impacted cell metabolism mainly when growth happened during VHEP experiments.

### Implication for scale up biomass production process

The understanding and control of biomass production processes present an important interest from an economical point of view. The main microorganism used for these processes is *Saccharomyces* because of its utilization in a wide variety of food industries. Van hoek *et al*. [32] showed that the quality of commercial baker’s yeast (*S*. *cerevisiae*) was determined by many parameters including storage, stability, osmotolerance, freeze-thaw resistance, rehydratation resistance and color.

Under aerobic conditions, the ethanol formation appeared when the residual glucose concentration was higher than 0.1 g L^-1^ for which the specific growth rate exceeded the critical value D_c_[4,33-35].

Nevertheless, this particularity is quite undesirable during biomass production since it reduces the biomass yield on the carbon feedstock. For this reason, industrial starter yeast production is performed in aerobic, sugar-limited fed-batch cultures. The optimization of process productivity therefore requires the higher increase as possible of the specific growth rate and / or of the biomass yield. Process optimization has been so far based on strain selection and empirical optimization of the environmental parameters such as pH, temperature, aeration rate and feeding profiles of sugar, nitrogen and phosphorus [36-38].

Moreover, large tanks used for industrial production lead to scale-up difficulties due to the appearance of heterogeneities, in particular for glucose and oxygen concentrations. Mixing time was estimated between 10 seconds and 250 seconds considering the tank size from 12 to 30 m^3^[39-42]. Mimicking the cell life in bioreactor was done with complex systems of bioreactors presenting different substrate and oxygen conditions. It revealed onsets of metabolic rearrangements in the order of the second [39,43]. The present study and previous work of Feria-Gervasio *et al*. [19] showed that addition of oleic acid in the fermentation broth permitted the yeast to present a delay for the metabolic shift onset after a sudden increase of glucose concentration in the range of few minutes.

Thus, an industrial use of oleic acid in such processes could lead to better manage the heterogeneities issues when the Crabtree effect onset is not desirable. Moreover, we demonstrated that the impact of oleate was not strain dependent but reversible. Therefore, starter yeasts prepared with oleate as co-substrate of glucose could be further used in various applications, implementing the Crabtree effect or not.

## Conclusions

In this work we demonstrated that adding oleic acid as a co-substrate of glucose allowed to decrease the occurrence of the “short-term” Crabtree effect as well as the “long-term” one. In both cases, biomass production was widely enhanced to the detriment of ethanol, and an 8% increase in the critical dilution rate has been reported. Because these biochemical phenomena were shown to be not strain dependent but reversible, industrial applications could be rationally envisaged in various purposes requiring a more efficient control of the Crabtree effect.

## Methods

### Strain and media

The yeast strain *S*. *cerevisiae* CEN.PK 113-7D [44] and CA10/pCD63 (provided by Sanofi Aventis industry) were stored in 30% glycerol at -80°C. CA10/pCD63 was auxotroph for adenine and histidine [45]. The cells were first grown on YPD plates (10 g L^-1^ yeast extract, 20 g L^-1^ bactopeptone, 20 g L^-1^ glucose, 15 g L^-1^ agar) at 30°C. All subsequent pre-cultures and culture experiments were carried out at 30°C in mineral medium as described by Feria-Gervasio *et al*. [19], except for fed-batch technique. When using the CA10/pCD63 strain the mineral medium was supplemented with adenine and histidine in the reservoir medium at 0.2 g L^-1^. Medium used for fed-batch experiment contained per litre: KH_2_PO_4_, 6 g; (NH_4_)_2_SO_4_, 12 g; MgSO_4_, 1 g. A sequential vitamins and trace elements feeding strategy based on the growth profile was applied [46] to reach per liter: EDTA, 0.03 g; ZnSO_4_.7H_2_O, 0.009 g; MnSO_4_.H_2_O, 0.002 g; CoCl_2_.6H_2_O, 0.0006 g; CuSO_4_.5H_2_O, 0.0006 g; Na_2_MoSO_4_.2H_2_O, 0.008 g; CaCl_2_.2H_2_O, 0.009 g; (NH_4_)_2_Fe(SO_4_)_6_.6H_2_O, 0.006 g; H_3_BO_3_, 0.002 g; D-biotin, 0.00024 g; D-L-pantothenic acid, 0.005 g; nicotinic acid, 0.005 g; myo-inositol, 0.125 g; thiamin, 0.005 g; pyridoxin, 0.005 g; para-aminobenzoic acid, 0.001 g.

### Chemostat cultivations with *S*. *cerevisiae* CA10/pCD63

Chemostat cultures with glucose as the sole carbon source and with glucose plus oleate were performed as described by Feria-Gervasio *et al*. [19]. The dilution rate was set at 0.18 h^-1^ for *S*. *cerevisiae* CA10/pCD63 to maintain the cells under pure oxidative metabolism. The bioreactors were fed with the mineral medium supplemented with glucose at 38 g L^-1^ for glucose chemostat and 39 g L^-1^ for glucose-oleate chemostat. This glucose-oleate chemostat was also fed with a 720 g L^-1^ oleic acid solution at a dilution rate of 0.0041 h^-1^.

After establishment of the steady state, six independent samples were taken over a period of 40 h to characterize it. Then glucose pulses were performed by injecting a known volume of a 600 g L^-1^ glucose solution in order to obtain a concentration in the reactor of 10 g L^-1^. The influent and effluent pumps were running continuously during the experiment. These experiments were carried out twice for each condition in two independent chemostats.

### Accelerostat cultivations with *S*. *cerevisiae* CEN.PK 113-7D

The accelerostat (A-stat) technique consisted of a computer-controled continuous cultivation procedure with a smooth change of the dilution rate [47]. Accelerostat cultures were performed with the CEN.PK 113-7D strain in the same conditions as for chemostat cultures, with a glucose concentration of 37 g L^-1^ for glucose A-stat and 38 g L^-1^ for glucose-oleate A-stat. The A-stats were launched when chemostat steady state cultures were stabilized at D_0_ = 0.16 h^-1^ by increasing linearly the dilution rate with a constant acceleration rate of α = 0.005 h^-2^, where D_t_ changed with time as follow:

$$D_{t}=D_{0}+\alpha *t$$

Where: D_t_ is the dilution rate for the instant “t”; D_0_ represents the initial dilution rate; α is the constant acceleration rate fixed and t the time in hours.

### Batch cultivations with *S*. *cerevisiae* CEN.PK 113-7D

Batch cultures were performed in a 5 L bioreactor B DCU B.Braun with a working volume of 3 L, managed with the MFCS/win 2.0 software. Temperature was regulated at 30°C and pH at 5.0 by addition of 1 M NaOH. Air flow and stirring rate were adjusted to maintain fully aerobic condition, i.e. a dissolved oxygen concentration above 20% of saturation. Initial glucose concentration was 40 g L^-1^, supplemented with oleic acid at 30 g L^-1^ for B_GO_C_GO_. Inoculation was carried out with *S*. *cerevisiae* CEN.PK 113-7D cells harvested at steady state in a glucose-limited chemostat at D = 0.16 h^-1^ for B_G_C_G_, or in a glucose-limited chemostat at D = 0.16 h^-1^ with oleate as co-substrate at D = 0.0073 h^-1^ for B_G_C_GO_ and B_GO_C_GO_[19] (Table 2).

**Table 2:** Summary of the different experiments carried out in the present study

**Process**	**Substrate**	**Cells**		**Acronym**
		**Strain**	**Preparation**	**Culture**_**C**. **source**_ / **Cells**_**C**. **source**_
Chemostat	Glucose	CEN.PK 113-7D	Glucose	
Chemostat	Glucose / Oleate	CEN.PK 113-7D	Glucose / Oleate	
Chemostat	Glucose	CA10/pCD63	Glucose	
Chemostat	Glucose / Oleate	CA10/pCD63	Glucose / Oleate	
Accelerostat	Glucose	CEN.PK 113-7D	Glucose	AS_G_ C_G_
Accelerostat	Glucose / Oleate	CEN.PK 113-7D	Glucose / Oleate	AS_GO_ C_GO_
Batch	Glucose	CEN.PK 113-7D	Glucose	B_G_ C_G_
Batch	Glucose	CEN.PK 113-7D	Glucose / Oleate	B_G_ C_GO_
Batch	Glucose / Oleate	CEN.PK 113-7D	Glucose / Oleate	B_GO_ C_GO_
Fed-batch	Glucose	CEN.PK 113-7D	Glucose	FB_G_ C_G_
Fed-batch	Glucose / Oleate	CEN.PK 113-7D	Glucose / Oleate	FB_GO_ C_GO_

Cells were previously prepared in glucose as sole carbon source (non adapted cells) or in glucose plus oleic acid (adapted cells).

### VHEP fed-batch cultivations with *S*. *cerevisiae* CEN.PK 113-7D

Fed-batch cultures were performed in a 5 L bioreactor B DCU B.Braun with a working volume of 3 L, managed with the MFCS/win 2.0 software. Temperature was regulated at 30°C and pH at 4.0 by addition of 14% (^v^/_v_) NH_3_ solution. Air flow and stirring rate were adjusted to maintain fully aerobic condition, i.e. a dissolved oxygen concentration above 20% of saturation. Initial glucose concentration was 100 g L^-1^ for all experiments, supplemented with oleic acid at 50 g L^-1^ for the glucose-oleate fed-batch. When glucose concentration reached 20 g L^-1^ in the bioreactor, pulses of a 700 g L^-1^ glucose solution was achieved to reach 100 g L^-1^. At the later phase of cultivation, i.e. when ethanol concentration was above 90 g L^-1^, the targeted glucose fed concentration was 50 g L^-1^. For glucose-oleate fed-batch (FB_GO_C_GO_), oleic acid feeding strategy was also performed to maintain the 50 g L^-1^. Inoculation was done from YPD plate of *S*. *cerevisiae* CEN.PK 113-7D. Three steps of propagation were carried out (5 mL, 30 mL and 300 mL) to inoculate the fed-batch cultures. Each preculture was grown for 10 hours at 30°C, 100 rpm and was used to inoculate the next-step at 10% (^v^/_v_) ratio. For glucose-oleate fed-batch cultivation, cells were firstly adapted to oleic acid by a growth phase in the bioreactor on 10 g L^-1^ trehalose, supplemented with 50 g L^-1^ of oleic acid. This strategy was carried out in order to mimic oxidative chemostat culture on glucose-oleate [48]. When trehalose was fully consumed, glucose was feed to reach 100 g L^-1^ and the culture was done as described above. Ethanol assessment was done as described by Pagliardini *et al*. [31] and taken into account for further calculations.

### Off-gas analysis

Inlet and outlet gases compositions were analyzed by mass spectrometry. For chemostats and A-stats, analyses were performed every 5 min during the steady state, every 45 s during A-stats acceleration and every 5 s during the pulse dynamic with a PRIMA 600s (VG gas, Manchester, UK). For batches and fed-batches, analyses were performed every 5 min with a Proline Dycor (Ametek Process Instrument, Berwyn, USA). Aeration started 1 hour after inoculation to avoid CO_2_ stripping from the medium and then prevent lag phase. CO_2_ production rate and O_2_ consumption rate were calculated as described by Poilpre *et al*. [49] for chemostats, A-stats and batches, and considering the liquid and gas volume evolution, the inlet airflow, the temperature and the pressure. The Respiratory Quotient (RQ) was given by the molar ratio between rCO_2_ and rO_2_.

### Determination of biomass

In order to conduct experiments, spectrophotometric measurements at 620 nm were performed with spectrophotometers Hitachi U- 1100 (Hitachi High Technologies America Inc., Schaumburg, USA) or Libra S4 (Biochrom, Cambridge, UK) after a calibration against cell dry weight measurements to evaluate yeast growth. For cell dry weight determination, culture medium was harvested and filtrated on 0.45 μm pore-size polyamide membranes (Sartorius AG, Göttingen, Germany), which were then dried to a constant weight at 60°C under partial vacuum (200 mmHg, i.e. approximately 26.7 kPa). In presence of oleic acid, 500 μl of culture medium were mixed with 500 μl of iso-propanol to eliminate oleic acid. The mixture was vortexed for 1 min and centrifuged for 3 min at 12.000×g. The pellet was resuspended in 500 μl of water for spectrophotometric measurements. For cell dry weight determination, membranes were washed after filtration with hexane and water to eliminate oleic acid. It was previously checked that iso-propanol and hexane did not damage the cells.

### Determination of cell viability

A cell coloration with the methylene blue method [50] was used as previously described to determine cells viability [46]. In presence of oleate, cell suspension was prepared as described above before staining procedure.

### Metabolites analysis

Sampling for metabolite analysis was performed by sampling the broth from bioreactors directly through a sterile 0.45 μm pore-size polyamide membranes (Sartorius AG, Göttingen, Germany). The permeate was then either directly analyzed (chemostats and A-stats) or frozen at -20°C for further analyses (batches and fed-batches).

In order to conduct experiments, glucose concentration was analyzed by enzymatic method with an YSI analyser model 27 A (YSI Life Science, Yellow Springs, USA). The accurate determination of glucose, ethanol, glycerol and organic acids from the permeate was performed by high-performance liquid chromatography (HPLC) as described by Alfenore *et al*. [46]. For chemostats and A-stats, the concentrations of ethanol and acetic acid were determined by gas chromatography as described by Feria-Gervasio *et al*. [19].

Oleic acid concentration determination was performed by two methods on the filtered supernatant obtained after iso-propanol washing and centrifugation. On the first hand, supernatant was directly injected in a HPLC using a 250*4.6 mm C18 column (Interchim, Montluçon, France). Column temperature was set at 50°C and a 3% (v/v) acetic acid in methanol solution was used as carrier with a flow rate of 1 ml min^-1^. Detection was done with a refractometer. On a second hand, supernatant was processed with a 0.2 mol L^-1^ trimethylsulphonium hydroxide in methanol solution in order to turn fatty acids into volatile molecule by methylation of their carboxilic function. Then sample was analyzed by gas chromatography using a 50 m*0.25 mm CP-Select CB for FAME fused silica WCOT (Varian, Palo Alto, USA). Injector temperature was set at 140°C and column temperature was initially set at 50°C, then be increased to 240°C with the following profile: 8°C min^-1^ for 3 min; 13°C min^-1^ for 5 min; 1.5°C min^-1^ for 27 min; 5°C min^-1^ for 12 min and a final isotherm of 10 min. Nitrogen gas was used as carrier with a flow rate of 50 ml.min^-1^ and detection was done by a FID set at 250°C.

## Competing interests

The authors declare that they have no competing interests.

## Authors’ contributions

JM, DFG and JRM worked equally on this project and should be considered all three as first authors. JM, DFG, JRM and SG contributed to the cell cultures experiments SG conceived of the study, and participated in its design and coordination and helped to draft the manuscript. All authors read and approved the final manuscript.
